# Microelectrode characterization of coral daytime interior pH and carbonate chemistry

**DOI:** 10.1038/ncomms11144

**Published:** 2016-04-04

**Authors:** Wei-Jun Cai, Yuening Ma, Brian M. Hopkinson, Andréa G. Grottoli, Mark E. Warner, Qian Ding, Xinping Hu, Xiangchen Yuan, Verena Schoepf, Hui Xu, Chenhua Han, Todd F. Melman, Kenneth D. Hoadley, D. Tye Pettay, Yohei Matsui, Justin H. Baumann, Stephen Levas, Ye Ying, Yongchen Wang

**Affiliations:** 1 School of Marine Science and Policy, University of Delaware, Newark, Delaware 19716, USA; 2 Department of Marine Sciences, University of Georgia, Athens, Georgia 30602, USA; 3 School of Earth Sciences, The Ohio State University, Columbus, Ohio 43210, USA; 4 Ocean College, Zhejiang University, Hangzhou 310058, China; 5 Department of Physical and Environmental Sciences, Texas A&M University – Corpus Christi, Corpus Christi, Texas 78412, USA; 6 Key Laboratory of Marine Bio-resources Sustainable Utilization, South China Sea Institute of Oceanology, Chinese Academy of Sciences, Guangzhou 510301, China; 7 ARC Centre of Excellence for Coral Reef Studies, School of Earth and Environment and UWA Oceans Institute, University of Western Australia, Crawley, Western Australia 6009, Australia; 8 School of Mechanical Engineering and Automation, Zhejiang Sci-Tech University, Hangzhou 310023, China; 9 Key Laboratory of Marine Ecosystem and Environment, State Oceanic Administration (SOA) Second Institute of Oceanography, Hangzhou 310012, China; 10 Reef Systems Coral Farm, New Albany, Ohio 43054, USA

## Abstract

Reliably predicting how coral calcification may respond to ocean acidification and warming depends on our understanding of coral calcification mechanisms. However, the concentration and speciation of dissolved inorganic carbon (DIC) inside corals remain unclear, as only pH has been measured while a necessary second parameter to constrain carbonate chemistry has been missing. Here we report the first carbonate ion concentration ([CO_3_^2−^]) measurements together with pH inside corals during the light period. We observe sharp increases in [CO_3_^2−^] and pH from the gastric cavity to the calcifying fluid, confirming the existence of a proton (H^+^) pumping mechanism. We also show that corals can achieve a high aragonite saturation state (*Ω*_arag_) in the calcifying fluid by elevating pH while at the same time keeping [DIC] low. Such a mechanism may require less H^+^-pumping and energy for upregulating pH compared with the high [DIC] scenario and thus may allow corals to be more resistant to climate change related stressors.

## Abstract (web summary)

Predicting coral response to ocean acidification is dependent on our understanding of their internal carbonate chemistry. Here, using microelectrodes, the authors show that corals elevate pH and carbonate ion concentration in their calcifying fluid, but keep total dissolved inorganic carbon low.

## Graphical abstract

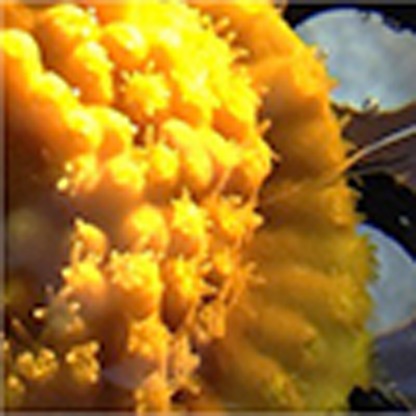

Atmospheric CO_2_ has increased from 280 parts per million (ppm) during pre-industrial times to 400 ppm today^1^. This increase has led to invasion of CO_2_ into the surface ocean, which has shifted the acid–base equilibrium and modified the dissolved inorganic carbon (DIC) species composition such that the proton concentration ([H^+^]) has increased, and pH and [CO_3_^2−^] have decreased; a process commonly known as ocean acidification (OA)^2^,^3^. Since OA reduces calcification rates in many, though not all, corals^4^,^5^,^6^,^7^,^8^, it is particularly detrimental to the health of coral reefs and their associated ecosystem functions and biogeochemical processes^4^,^7^,^9^. The decline in coral calcification rates is thought to be linked to reductions in seawater [CO_3_^2−^] and thus *Ω*_arag_. However, because calcification occurs in a semi-isolated compartment where conditions are largely controlled by the coral^10^, the exact mechanism by which OA reduces calcification is difficult to decipher^11^. To understand and predict how corals might respond to current and future CO_2_ increases, climate warming and other stressors, we need a better understanding of coral calcification mechanisms, particularly how a high pH and *Ω*_arag_ state is achieved inside the calcifying fluid and the sources as well as speciation and concentration of DIC that sustain calcification^5^,^11^,^12^,^13^,^14^,^15^,^16^.

In order to precipitate CaCO_3_ in a place separated from seawater by multiple layers of tissue, corals ultimately must be able to import Ca^2+^ ions and DIC from seawater and transport them to the site of calcification. These processes remain poorly understood and controversial. Although proposed routes of ion and molecule import differ in their details, potential import processes can be broadly grouped into three categories: (1) passive CO_2_ diffusion, (2) active bicarbonate and other ion transport and (3) whole seawater import though the time scale of each of these processes will differ^10^,^16^,^17^,^18^,^19^. Which of these three processes dominates the import pathway for DIC species remains unknown in part because a complete characterization of the internal carbonate chemistry of corals is lacking. In addition to the import of necessary ions, corals must raise the aragonite saturation state of the calcifying fluid to obtain rapid rates of calcification^18^. It is widely believed that this is achieved through a H^+^ efflux pump that creates high pH and, via chemical equilibrium and kinetic limitations, high [CO_3_^2−^] in the calcifying space^5^,^19^. The H^+^ efflux pump is proposed to be coupled to Ca^2+^ influx via a Ca–H ATPase^10^. This theoretical model for coral calcification is considered the foundation for hypotheses explaining the response of calcification to future acidification^5^, yet direct measurements of at least two of the several carbonate system parameters to fully substantiate this conceptual model for coral calcification are lacking. Based on either indirect geochemical tracer evidence^12^ or sensor-based pH data combined with the assumption of either active HCO_3_^−^ transport or whole seawater import^13^,^20^, several studies have also suggested that corals concentrate DIC to roughly twice the seawater value at the site of calcification. Others, however, have assumed that coral internal [DIC] is similar to or lower than that in seawater^5^,^21^. Furthermore, while a linkage between H^+^-pumping and carbon species transportation has recently been suggested^12^, confirmation by direct measurements is required. In summary, despite decades of research, our knowledge of the internal carbonate chemistry of corals is still limited and direct evidence is needed to definitively constrain coral calcification mechanisms.

Microelectrodes are a powerful tool for the direct measurement of chemical concentrations within organisms. The reliability and accuracy of pH and other microelectrodes have been well-established in the literature and in our work (see Methods section below). Previous pH microelectrode studies have shown that pH increases near the coral surface and in the upper region of the polyp's gastric cavity due to photosynthesis, but decreases deeper in the cavity (coelenteron)^22^. Most importantly, a high pH has been measured in the calcifying fluid using both pH microelectrodes (pH: 9.3–10.1) and pH sensitive dyes (pH: 8.5–9.0) (refs 5, 13, 23). These experiments showed that corals increase the pH of the calcifying fluid to achieve a high *Ω*_arag_, which facilitates rapid rates of calcification. However, to fully characterize internal coral carbonate chemistry, pH alone is not sufficient and another carbonate system parameter such as [CO_3_^2−^], [DIC], or total alkalinity (TA) must be assumed^12^,^13^ or preferably directly measured.

Here we use CO_3_^2−^ microelectrodes together with pH microelectrodes to directly measure two necessary system parameters and calculate concentrations of all other carbonate species within corals. Our novel findings are that [CO_3_^2−^] and carbonate saturation state inside the coral calcifying fluid are high and calcifying fluid DIC concentration is low.

## Results

### pH and [CO_3_^2−^] depth profiles

**Figure 1:**
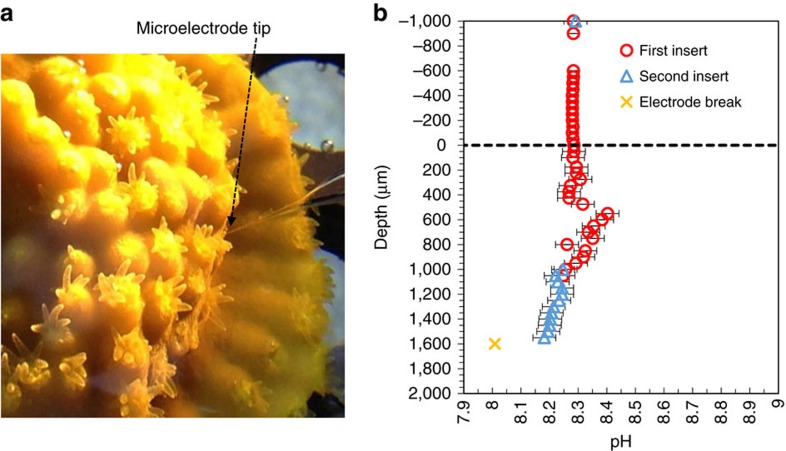
Microelectrode profiling and electrode performance. (**a**) Close-up view of the coral *T. reniformis* showing the tip of a microelectrode as it enters the polyp. (**b**) Repeated pH microelectrode profile readings inside a *T. reniformis* coral polyp. Between the last point of the second insert and the next reading (which is a noise-free reading and is marked as ×), the microelectrode tip broke. pH microelectrodes were calibrated on the NBS scale, which is about 0.1–0.15 unit greater than that on the total scale. Error bars (s.d.) are defined in the Methods section. Positive depths are inside the coral polyp while negative depths are in the overlying seawater. Error bars are smaller than the symbols in the overlying water.

pH and CO_3_^2−^ microelectrodes were used to characterize the interior of coral polyps (see Methods section below) under ambient seawater pH and in the light (500 μmol photons m^−2^ s^−1^), when photosynthesis stimulates high rates of calcification. We advanced a microelectrode inside the polyp's gastric cavity at a resolution of 50 μm per step (5 μm in one case) parallel to the polyp wall until it broke on the solid CaCO_3_ base (Fig. 1a,b). Repeated micro-profile measurements from seawater into the lower part of the coelenteron revealed that the profiles were reproducible (Fig. 1b, Supplementary Figs 1a,b and 2) even though polyp contraction sometimes affected the observed depths (Supplementary Fig. 2) and introduced noise in the electrode data.

**Figure 2:**
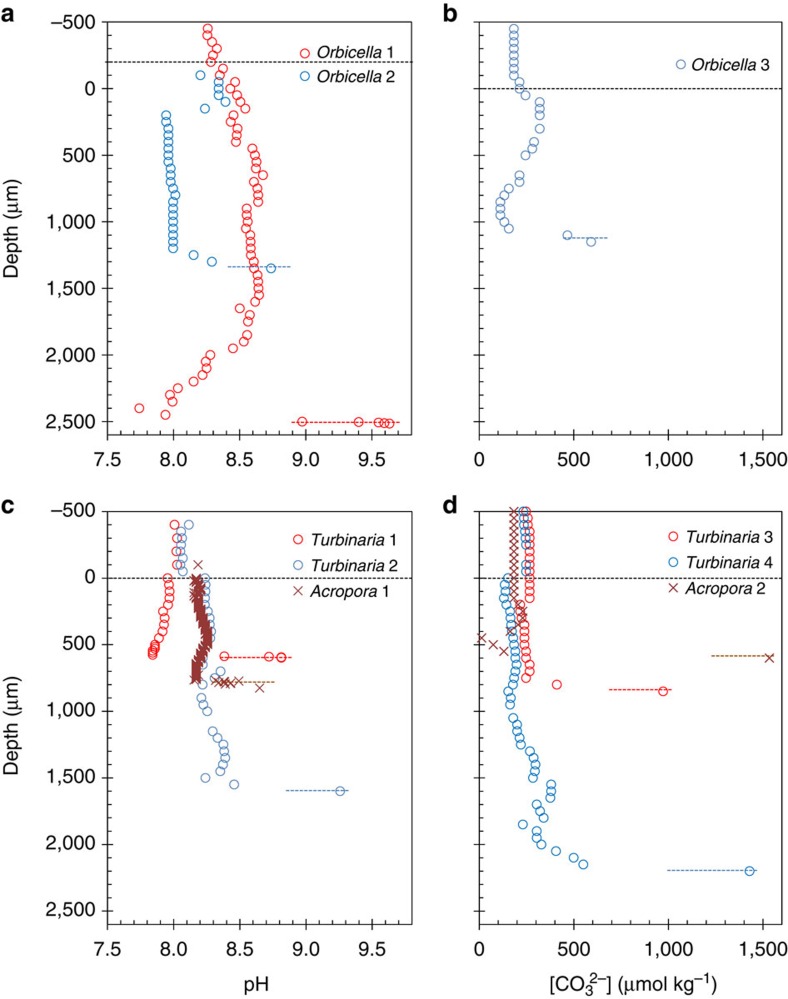
Coral internal pH and [CO_3_^2−^] profiles through the polyp mouths. (**a**) pH and (**b**) [CO_3_^2−^] of *Orbicella faveolata* corals. (**c**) pH and (**d**) [CO_3_^2−^] of *Turbinaria reniformis* and *Acropora millepora* corals. Dashed black lines indicate the top of the polyp mouths. Colored dashed lines roughly indicate the location of the calcifying fluid and differ in depth for each polyp.

We obtained several full-depth profiles of pH and CO_3_^2−^ through the polyp mouth, the coelenteron, and into the calcifying fluid for three tropical coral species: *Orbicella faveolata, Turbinaria reniformis* and *Acropora millepora*. For *O. faveolata*, pH (in National Bureau of Standards, NBS, scale) increased from 8.1 and [CO_3_^2−^] from 180 μmol kg^−1^ in the overlying seawater to 8.3–8.5 and ∼300 μmol kg^−1^, respectively, in the upper part of the polyp due to photosynthesis (Fig. 2a,b). Deeper in the polyps the pH decreased to a value of 7.7, which was consistent with earlier studies^13^,^22^,^24^. Correspondingly, in the deeper portion of the polyp, [CO_3_^2−^] decreased to 100 μmol kg^−1^. Then, within a short distance, pH increased sharply to as high as 9.7, confirming previous work^5^,^13^,^23^, while [CO_3_^2−^] increased six fold to 600 μmol kg^−1^ before the electrode tip broke. We interpret this rapid increase in pH and [CO_3_^2−^] as evidence that the microelectrodes obtained measurements within the calcifying fluid prior to breaking.

Similar profiles were measured inside coral polyps of *T. reniformis* with highest pH in the calcifying fluid ranging from 8.8 to 9.3 (Fig. 2c) and highest [CO_3_^2−^] from 1000 to 1400 μmol kg^−1^ (Fig. 2d). In *A. millepora*, the electrode advancement step was reduced to 5 μm due to the smaller polyps compared with the other species, resulting in a very fine-scale pH profile, which demonstrated a sharp increase in pH from 8.15 to 8.65 within only a short distance of 10–60 μm (Fig. 2c). Similarly, we observed a 15-fold increase in [CO_3_^2−^] from ∼100 μmol kg^−1^ in the coelenteron to ∼1500 μmol kg^−1^ in the calcifying fluid within a distance of 50 μm in this species (Fig. 2d).

### Composite depth profiles of CO_2_, DIC, TA and *Ω*_arag_

Our methods did not allow simultaneous profiling of pH and [CO_3_^2−^] at the same spots due to both technical challenges and high heterogeneity inside a coral polyp. Therefore, we constructed composite profiles of pH and [CO_3_^2−^] based on the range of our data across all three species and calculated [CO_2_], DIC, TA and *Ω*_arag_ profiles (Supplementary Table 1), assuming the DIC system was in chemical equilibrium (see Methods section below). These composite profiles were then used to generate a more complete picture and a conceptual model of coral calcification.

**Figure 3:**
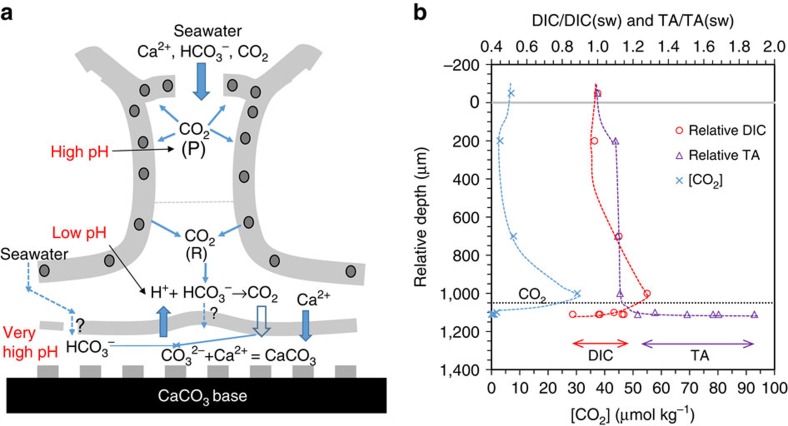
Conceptual model of coral calcification and CO_2_ transport mechanism and distributions of internal [CO_2_] and relative DIC and TA to seawater values. (**a**) Illustrative model and (**b**) concentration distributions. Dark circles are photosynthetic endosymbionts. P stands for photosynthesis, R for respiration, and sw for seawater. In (**b**), for DIC (red line) and TA (purple line) the calculated ranges of possible concentrations inside the calcifying fluid are provided based on calculations of all species from Supplementary Table 1.

Overall, our study shows that DIC decreases slightly near the polyp surface and in the upper part of the coelenteron due to photosynthetic CO_2_ consumption (Fig. 3a,b). Farther down in the polyp calculated DIC increases due to respiratory input of CO_2_ and reaches a peak at the bottom of the coelenteron before rapidly declining again in the calcifying space (Fig. 3b). Calculated TA in the coelenteron is nearly constant throughout most of the gastric cavity space, but increases dramatically in the calcifying fluid (Fig. 3b). Nonetheless, in agreement with direct TA measurements of the coelenteron fluid^25^ and some model assumptions^5^,^21^, DIC and TA values in the coelenteron solution are not substantially different from those of the overlying seawater.

Notably, a sharp increase in [CO_2_] to ∼30 μmol kg^−1^ occurs toward the bottom of the coelenteron solution (Fig. 3b). This increase is not only due to an increase in DIC from respiration but is substantially enhanced by the nearly invariable TA (Fig. 3b, Supplementary Table 1). Thus, we attribute this [CO_2_] increase to both respiration input and the conversion of CO_3_^2−^ and HCO_3_^−^ to CO_2_ by H^+^ exported from the calcifying fluid (Fig. 3a) – a mechanism suggested earlier as being important for *Symbiodinium* spp. photosynthetic needs^19^. Inside the calcifying fluid [CO_2_] is nearly zero (Fig. 3b), primarily due to the fluid's very high pH (Fig. 2a,c). This very large [CO_2_] difference (∼30 μmol kg^−1^, Supplementary Table 1), based on direct measurements for the first time, confirms recent indirect evidence from geochemical tracers^12^ that a high rate of CO_2_ molecular diffusion from the coelenteron through the tissue into the calcifying fluid provides an important source of DIC for CaCO_3_ precipitation.

### CO_2_ flux into the calcifying fluid

We estimated the CO_2_ influx to the calcifying fluid by both a 1-D diffusion model and an exchange model between the coelenteron and the calcifying fluid^21^,^26^ (equations and parameters are given in the Methods section and Supplementary Table 1). The CO_2_ diffusive flux was estimated to be as high as 217–433 nmol cm^−2^ h^−1^. Alternatively, the CO_2_ influx was estimated by the exchange model to be 82–301 nmol cm^−2^ h^−1^ (see Methods section). This range of CO_2_ influx and consumption rate compares favourably with coral calcification rates measured in the past (150–1000, nmol cm^−2^ h^−1^) (refs 10, 27). Based on the observations and model calculations presented here, we postulate that the coral's proton pumping ability is essential not only for creating a high aragonite saturation state in the calcifying fluid (favouring calcification), but also for creating a sharp CO_2_ gradient facilitating DIC influx to support calcification. This CO_2_ transport mechanism is consistent with dual isotopic evidence^18^,^28^ and geochemical proxies^12^ showing that respired CO_2_ is an essential source of DIC for calcification. This passive CO_2_ transport mechanism may reduce the need for the active transport of HCO_3_^−^ by processes such as transmembrane or vacuolar transport though the ultimate source of DIC for calcification comes from external seawater via photosynthesis, diffusive flux and whole seawater import.

### Low [DIC] in the calcifying fluid

Despite the great CO_2_ flux into the coral calcifying fluid, our analysis reveals that the fluid [DIC] is not that different from seawater concentrations due to the consumption of DIC during calcification, which balances the CO_2_ influx (Fig. 3a). Even though TA in the calcifying fluid is elevated (1.2–1.9 times that of overlying seawater) it is not as high as previously reported values^12^. Allison *et al*.^12^ have recently used geochemical tracers to determine pH (from B isotope ratio) and estimate DIC (from B/Ca ratio) and concluded that in order to achieve a high aragonite saturation state, corals must internally concentrate DIC. Others also assumed that corals concentrate internal DIC to about twice, or more, the concentration of seawater^13^,^29^. Our direct pH and [CO_3_^2−^] measurements show that while *Ω*_arag_ inside the calcifying fluid is elevated, DIC is not elevated with respect to background seawater.

## Discussion

Our microelectrode profiles of pH and CO_3_^2−^ through coral polyps provide the first direct evidence for elevated [CO_3_^2−^] in the calcifying fluid. [CO_3_^2−^] (600–1500 μmol kg^−1^) and corresponding aragonite mineral saturation states (*Ω*_arag_=8–22) in the calcifying fluid are high and consistent with expectations based on inorganic CaCO_3_ formation experiments^11^, previous experiments^12^ and model frameworks^5^,^13^.

Data from the calcifying fluid was only obtained in a small subset of the numerous profiles collected, most likely because the calcifying fluid layer is very thin (likely only a few micrometers) and existed in pockets^13^,^16^,^17^. In most cases, the electrode tips broke within one step (50 μm) between the bottom of the polyp gastric cavity space (coelenteron) and the hard CaCO_3_ skeleton. In some cases, however, the data suggest the space between the coelenteron and the skeleton could be thicker than previously thought (up to 100 μm). Alternatively, it is possible that the newly formed CaCO_3_ minerals are porous allowing the glass microelectrode tip to slightly penetrate the mineral surface, thus giving an inaccurate measure of the calcifying fluid thickness. It would be desirable to collect profiles with a better constraint of vertical and lateral heterogeneity of carbonate properties inside corals. Nonetheless a high pH, high [CO_3_^2−^] layer was unequivocally detected right above the CaCO_3_ skeleton, though further work is needed to better define its thickness and assess its spatial variability. While the pH upregulation in the calcifying fluid measured by microelectrodes does overlap with those by the dye-based method^13^ and boron isotope method^12^, the highest values have been obtained from microelectrodes (9.7 here, 9.3 and 10.1 by two others, see Table 1)^5^,^23^. Differences between the approaches may result from differences in coral species, colony size^30^, measurement condition and location within the colony^13^,^14^, and time-scales over which the approaches integrate^30^.

**Figure 4:**
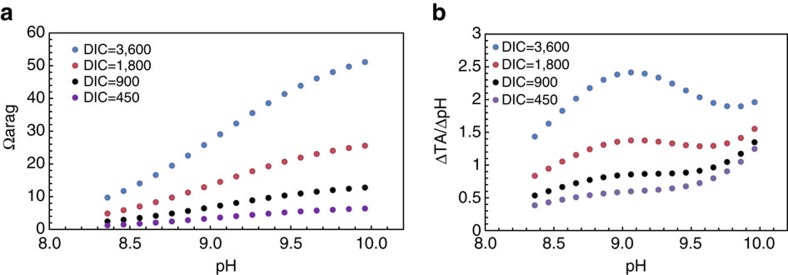
Aragonite mineral saturation state (*Ω*_arag_) and buffer index in the coral calcifying fluid as functions of pH and [DIC]. (**a**) *Ω*_arag_ and (**b**) buffer index. The buffer index is given as mmol kg^−1^ of H^+^ pumping (H^+^ loss or TA gain) per unit of pH upregulation. This index is calculated numerically with CO2SYS (ref. 37) (see Methods section). Here [DIC]=1800 μmol kg^−1^ is roughly the same as seawater value. pH is on the NBS scale.

If the very high pH of 9.0 or higher measured by microelectrodes reported here or elsewhere is typical of coral calcifying fluid, a high DIC internal solution (2 × of seawater DIC or higher) would indicate that internal *Ω*_arag_ could be as high as 30 to 55 (Fig. 4a), which is inconsistent with *Ω*_arag_ values inferred from abiotic experiments^18^. Therefore, it seems that the most reasonable internal [DIC] is about 1 × of seawater value, which is consistent with our results from the two direct carbonate parameter measurements. While further research is required to confirm our conclusion, our findings strongly shift the argument towards one of a lower calcifying fluid DIC concentration^5^,^21^.

One important difference between low versus high DIC scenarios is the necessary amount of protons that a coral must pump out (which results in an increase in TA) to raise its internal pH by one unit (in mmol-H^+^ per kg per pH unit). This buffer index for the high DIC scenario is at least twice that for the low DIC scenario, thus more protons must be removed in a high DIC scenario to achieve the observed calcifying fluid pH (Fig. 4b). Therefore, we conclude that the low DIC scenario would require less energy than the high DIC scenario as proton-pumping requires ATP. Comparisons of a relative change in energy requirement to upregulate internal pH between high external CO_2_ under future OA conditions and 400 ppm current conditions following the method of Ries^5^ also supported our conclusion (Supplementary Note 1 and Supplementary Table 5). As a result, if proton removal from the coelenteron is increasingly difficult as a result of higher seawater H^+^ concentrations under OA conditions^31^, the low DIC scenario should be advantageous as less H^+^-pumping will be required to further raise pH in the calcifying fluid.

Overall, this suggests that corals that use passive CO_2_ diffusion as an important pathway for DIC supply, and maintain low DIC in their calcifying fluid, may require less energy to calcify at high rates, which could potentially make them more resistant to OA and other stressors. However, at the same time, the lower buffering capacity of low DIC and moderate TA calcifying fluid means that the ability to maintain high fluid pH will be vulnerable to changes in the physiological and biochemical conditions that control H^+^-pumping. For example, a weakening of proton pumping during dark periods may lead a coral with low internal DIC towards decalcification more readily than otherwise expected in a high DIC scenario. In addition, a high internal DIC could allow corals to reach a high carbonate saturation state at lower internal pH (Fig. 4a)^13^. Therefore, additional studies using microelectrodes and other techniques in corals under OA conditions in combination with other stressors and environmental conditions (i.e., elevated temperature, nutrient levels and daytime versus nighttime) are a critical next step in elucidating coral calcification mechanisms under future ocean conditions.

## Methods

### Coral collection and preparation

The measurements focused on three species of corals: *O. faveolata, T. reniformis* and *A. millepora*. All species contain photosynthetic endosymbionts, often called zooxanthellae (*Symbiodinium* spp.), and form an aragonite skeleton. Small fragments of *O. faveolata* were sampled from the Florida Keys and transported to the laboratory in the University of Georgia (Florida Keys National Marine Sanctuary permit 2014–2015). *Turbinaria reniformis* and *Acropora millepora* coral fragments were provided by Reef Systems Coral Farm in New Albany, OH. The parent colonies of both species have been maintained in recirculating indoor aquaria with natural light (greenhouse, 700–1000 μmol photons m^−2^ s^−1^) and commercially available artificial seawater (Instant Ocean Reef Crystals) for over 10 years. Several fragments of each colony were shipped to the Cai lab at the University of Georgia, where they were acclimated to a closed-system laboratory aquarium with artificial seawater of salinity 35 and TA of 2.4 mmol kg^−1^ at 26 °C with light levels of about 200 μmol photons m^−2^ s^−1^ for at least two weeks prior to conducting pH and CO_3_^2−^ microelectrode profile measurements. TA in the aquarium was depleted at the time of microelectrode measurements (Supplementary Tables 2, 3 and 4) but was amended weekly.

### Microelectrode construction and properties

pH microelectrodes^32^,^33^ and CO_3_^2−^ microelectrodes^34^,^35^ were constructed as previously described. We made our microelectrodes with a flat tip of diameter between 10 and 15 μm (Supplementary Fig. 3), which enabled us to make pH measurements along a profile into the coral polyp. pH microelectrodes were calibrated with three commercial pH NBS standards (pH=4, 7 and 10) at 26 °C to determine their response slopes. CO_3_^2−^ microelectrodes were calibrated in CO_3_^2−^ standard solutions pumped sequentially into a sealed chamber where an electrode was hosted^35^. Carbonate concentrations were set at approximately 50, 150, 250, 350, 500 and 1,000 μmol kg^−1^ by adding controlled amounts of HCl and NaHCO_3_ into filtered seawater collected from the Gulf of Mexico^35^. Standard solutions were then bubbled with room air for 24 h to minimize further exchange of CO_2_ with air during the experiment. Finally, CO_3_^2−^ concentrations were precisely calculated by CO2SYS with TA and pH measured by Gran titration and a commercial Ross glass electrode, respectively.

### Data collection

In brief, a coral fragment was transferred from the incubation tank together with the tank water into a small measurement chamber that was kept at 26 °C and stirred with a magnetic stirrer (Supplementary Fig. 4). The coral fragment was allowed to acclimate in the small container for 1 h under 500 μmol photons m^−2^ s^−1^ until the microelectrode reading was stable in the chamber water (Fig. 1b & Supplementary Fig. 1). The slopes of pH and CO_3_^2−^ microelectrodes were calibrated immediately before use, and chamber water pH measured by a commercial electrode and measured TA values were used as the reference point before beginning the profiles. Measurement conditions are provided in Supplementary Tables 2,3 and 4. The point at which the microelectrode entered the polyp (defined as depth=0) was observed under a microscope. Typically the coral reacted by closing its mouth making this point unambiguous. The electrode was then advanced through the polyp's gastric cavity in increments of 5–50 μm until the microelectrode broke on the skeleton. The microelectrode was held at each step until a stable reading was obtained. The pH reading was considered stable when drift was <0.2 mV or 0.003 pH min^−1^ (or <0.2 mV min^−1^ for CO_3_^2−^) or a total of 3 min had passed, whichever was first. A 3-min limit was established because our pH microelectrodes had a much shorter response time^32^,^35^ but, in some cases, a stable reading was not realistic due to polyp movement.

Several replicate profiles were obtained to ensure that the microelectrodes were not fouled or otherwise damaged by profiling through the corals. In these tests a profile was obtained about half way through the coral coelenteron, at which point the electrode was returned to the overlying seawater, and the seawater readings were compared with values obtained before profiling was started. Then, the profile was resumed, repeating a portion of the earlier profile to ensure agreement between the two (Fig. 1b and Supplementary Figs 1 and 2). However, this practice cannot be extended into the calcifying fluid as an electrode is destined to be damaged and a repeating profile cannot be collected.

Finally, based on electrode calibration result, stability test and repeated measurements as shown above, we assign errors as ±0.02 pH unit for the overlying seawater, ±0.04 pH unit for the coelenteron and ±0.1 pH unit for the calcifying fluid. For [CO_3_^2−^], a maximum uncertainty of ±10% is established based on our experiences and literature^35^,^36^.

### Carbonate chemistry calculation

While the CO2SYS program^37^ is popularly used for carbonate equilibrium calculation based on two of the four parameters (TA, DIC, pH and *p*CO_2_), it does not accept the pH and [CO_3_^2−^] pair as input parameters. However, measured pH and [CO_3_^2−^] data allowed us to calculate concentrations of other DIC species, [DIC] and the TA in a straightforward way. For example,


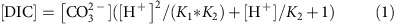


where, *K*_1_ and *K*_2_ are the dissociation constants of the carbonic acid^38^. All equations are described in Supplementary Note 2. As in previous studies, we assumed that the coral's internal salinity was the same as that of the external seawater salinity (that is corals are osmoconformers) for our thermodynamic calculation^5^,^12^,^21^. While a lower internal salinity would lead to a higher calculated DIC from the same observed pH and [CO_3_^2−^], for example, calculated [DIC] is 0.8–12% higher at salinity=30 than that at 35, conclusion derived here holds well within a reasonable salinity range (see more details in Supplementary Note 3). In this work, pH in NBS scale is used. However, when needed, pH in total proton scale is converted to NBS scale roughly by adding 0.15 unit, pH_NBS_=pH_T_+0.15, according to CO2SYS simulation^37^ and Dickson^39^.

Although internal [Ca^2+^] was reported to be about 10% higher than that in seawater^23^, as it was the only reported value and as seawater [Ca^2+^] is very high (∼10.3 mM), we assumed that [Ca^2+^] in the calcifying fluid is the same as in seawater in calculating the saturation state *Ω*. Since [CO_3_^2−^] is directly measured in this work, the uncertainty in *Ω* calculation is about the same as that in [Ca^2+^], roughly within 10%, and it does not affect the conclusions and discussion of this work.

### Calculation of CO_2_ flux into the calcifying fluid

In a 1-D diffusion model, CO_2_ molecular diffusional flux is defined as:


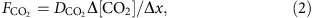


where, *D* is the diffusion coefficient, and Δ*x* is the distance corresponding to the concentration gradient. We assume *D* determined in seawater^40^ is applicable to the coral interior space. We calculated CO_2_ flux using the Δ[CO_2_] (Supplementary Table 1) and the corresponding distance between the two data points in the coelenteron and the calcifying fluid, respectively, as observed with our microelectrode (50–100 μm).

Using the calculated [CO_2_] gradient (29.8 μmol kg^−1^) from Supplementary Table 1, CO_2_ flux (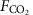) from the coelenteron into the calcifying fluid was calculated. Note the [CO_2_] gradient determined by the microelectrode data is not affected much by the uncertainty caused by relatively large pH and [CO_3_^2−^] ranges inside the calcifying fluid as [CO_2_] is nearly 0 (that is an uncertainty of [CO_2_] between 0.5 and 0.1 μmol kg^−1^ only changes [CO_2_] gradient from 29.9 to 30.3 μmol kg^−1^) (Supplementary Table 1). In other words, a conservative internal pH value of 9.1 and a high value of 9.5 lead to similar estimates of CO_2_ gradient and flux.

Flux can also be calculated as the exchange rate between two adjacent ‘boxes', the coelenteron and the calcifying fluid:


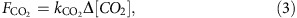


where, *k*co_2_ is the exchange coefficient (0.76–2.8 cm s^−1^) across biological tissues determined by an isotopic exchange method^41^, which has been used in two recent coral CO_2_ flux model simulations^21^,^26^. In principle, the two coefficients are linked by:


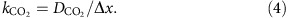


A lower CO_2_ flux calculated based on equation 3 than equation 2 may indicate that the CO_2_ diffusion coefficient across the coral tissue (or the coral tissue's permeability) is lower than that of the seawater.

### Amount of H^+^-pumping per unit pH upregulation

We define this parameter based on the buffer factor concept, discussed in details by Frankignoulle^42^, Hofmann *et al*.^43^ and Egleston *et al*.^44^. We take the buffer factor *β* defined below as a measure of how strong the calcifying fluid can buffer its pH:


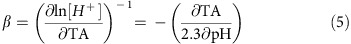


We rearranged the definition to ΔTA/ΔpH=−2.3*β*, which represents the amount of proton pumping (or TA gain) a coral must do to upregulate its calcifying fluid pH by one unit. This was calculated numerically by following the same approach as the Revelle Factor calculation in the CO2SYS program^37^. We first set four DIC values and for each DIC value we allowed a range of pH changes. Thus, we derived TA values for the various DIC and pH combinations. Then, a ±1 μmol kg^−1^ disturbance is applied to TA values and the corresponding perturbation in pH is derived using the TA and DIC pairs as the input parameters in CO2SYS (Fig. 4b).

### Energy consumption for pH upregulation

We calculated the relative energy changes of pH upregulation between different OA conditions as specified by Ries^5^ using the Nernst equation. The equations and results are given in the Supplementary Note 1 and Supplementary Table 5.

## Additional information

**How to cite this article:** Cai, W-J. *et al*. Microelectrode characterization of coral daytime interior pH and carbonate chemistry. *Nat. Commun.* 7:11144 doi: 10.1038/ncomms11144 (2016).

## Supplementary Material

Supplementary Information Supplementary Figures 1-4, Supplementary Tables 1-5, Supplementary Notes 1-3 and Supplementary References

## Figures and Tables

**Table 1:** Comparison of pH values measured in coral calcifying fluid by microelectrodes and other methods.

Technique	Reported by	Coral species	Light condition	Location	pH_T_	pH_NBS_	Notes
Microelectrode	Al-Horani *et al*.^23^	*Galaxea fascicularis* (tropical coral)	Light	Apexes		9.28	Sensor buried under tissue
Microelectrode	Ries^5^	*Astrangia poculata* (temperate coral)	Light	Apexes, between septal ridges		10.1	Through a predrilled incision
Microelectrode	This work	*Orbicella faveolata* (tropical coral)	Light	Apexes, under polyp mouth		8.75–9.65	Sensor penetrated via the mouth
Microelectrode	This work	*Turbinaria reniformis* (tropical coral)	Light	Apexes, under polyp mouth		8.8–9.3	Sensor penetrated via the mouth
Microelectrode	This work	*Acropora millepora* (tropical coral)	Light	Apexes, under polyp mouth		8.65	Sensor penetrated via the mouth
pH sensitive dye	Venn *et al*.^13^	*Stylophora pistillata* (tropical coral; microcolonies)	Light	Distal margin (edge)	8.55–8.85	8.70–9.00	Laterally grown on slide
B-isotopes	Allison *et al*.^12^	*Porites* spp.(tropical coral)	Light/dark cycle	Average	∼8.5	∼8.65	See citations therein

Note that only one B-isotope based result is given for comparison but many more are cited in the reference. Note, we convert pH_T_ to pH_NBS_ by adding 0.15.
