# Supplementary material for: Microelectrode characterization of coral daytime interior pH and carbonate chemistry
Source: Nat Commun. 2016 Apr 4;7:11144. doi: 10.1038/ncomms11144 (PMC4821998; doi:10.1038/ncomms11144)
Supplement: Supplementary Information — Supplementary Figures 1-4, Supplementary Tables 1-5, Supplementary Notes 1-3 and Supplementary References [file ncomms11144-s1.pdf]

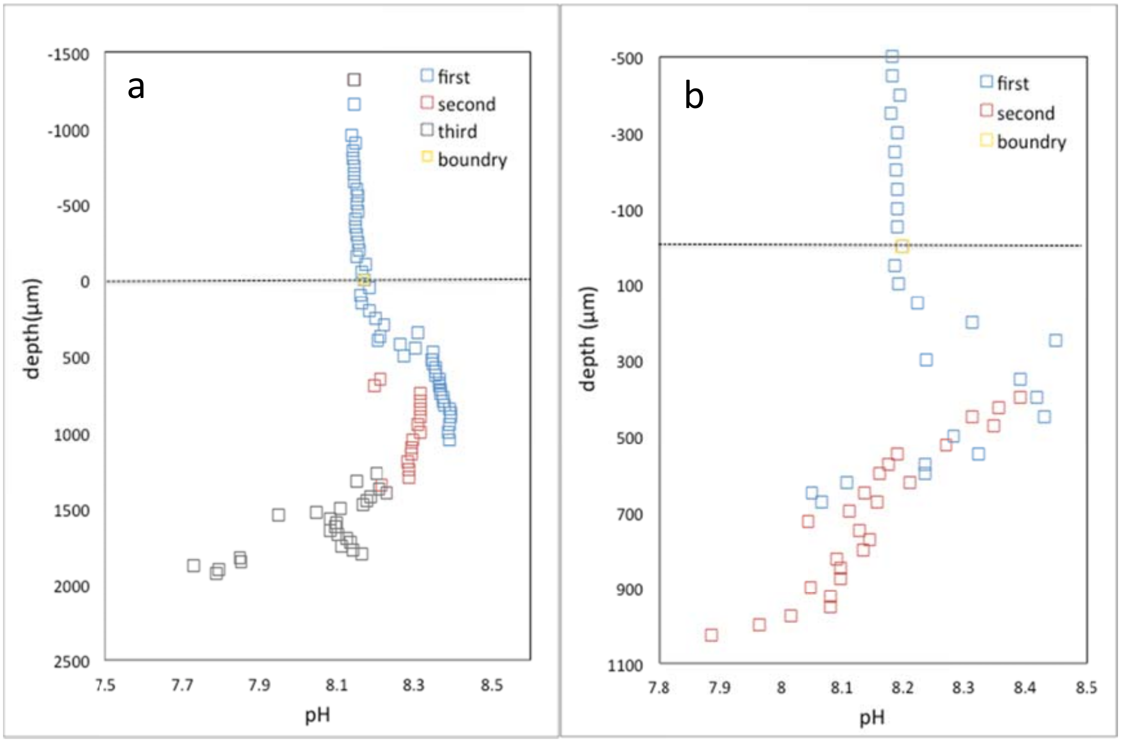

Supplementary Figure 1. pH microelectrode electrode performance and stability test, showing repeated pH_{NBS} microelectrode profile readings inside coral polyps. (a) *T. reniformis* and (b) *A. millepora*. Repeated profiles are listed as first (blue), second (red), and third (gray). The boundary between the coral mouth and seawater is shown with a dashed line bisected by a yellow square.

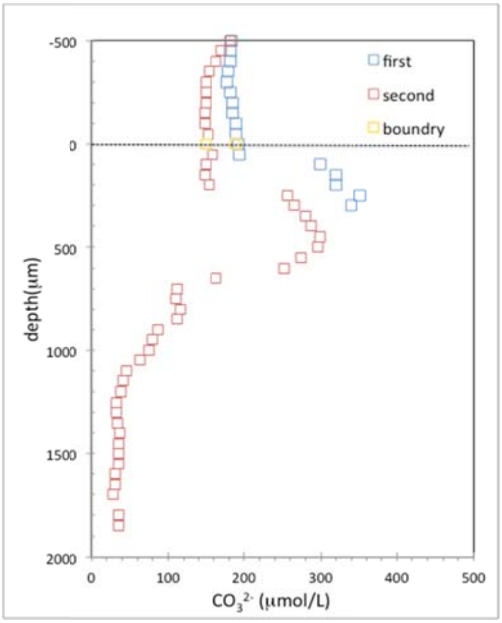

Supplementary Figure 2. Duplicate CO_3^{2-} microelectrode profiles on *T. reniformis.* At the second insert, the polyp contracted, and thus the interface moved down. If the depths are adjusted, the two profiles are highly repeatable. Repeated profiles are listed as first (blue) and second (red). The boundary between the coral mouth and seawater is shown with a dashed line bisected by a yellow square.

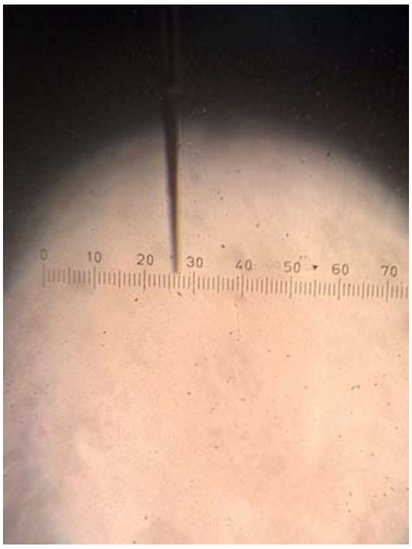

Supplementary Figure 3. pH microelectrode with a tip diameter between 15-20 μm. The microelectrode was photographed at 400x under a dissecting microscope. Note the microelectrode tip is flat and thus the vertical sensing resolution is believe to be only a few μm.

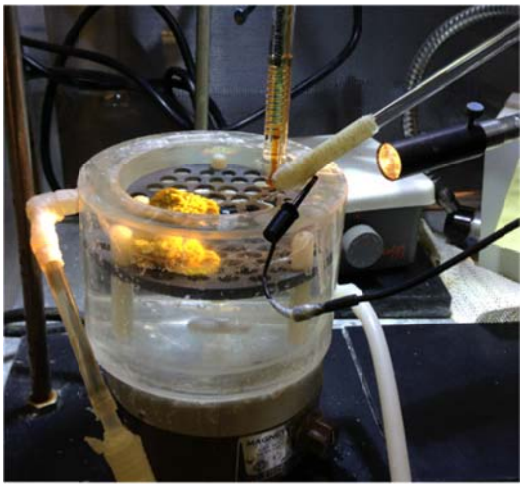

Supplementary Figure 4. Coral microelectrode setup with the coral *Turbinaria reniformis* in the seawater chamber.

Supplementary Table 1. pH, [CO_3^{2-}], DIC, TA, [CO_2] (in µmol kg^{-1}), pCO_2, and Ω_{arag} values inside an average coral polyp

Zone	pH	[CO_3^{2-}]	DIC	TA	[CO_{2aq}]	pCO_2	Ω_{arag}
Seawater	8.2	183	1479	1759	6.95	252	2.92
Upper coelenteron	8.5	320	1453	1937	3.05	111	5.10
Bottom coelenteron	**7.7**	**80**	**1893**	**1982**	**30.4**	**1100**	**1.28**
Likely combinations of the calcifying fluid	8.7	500	1616	2330	1.90	68.7	7.97
	8.9	700	1685	2649	1.06	38.3	11.2
	**9.1**	**900**	**1699**	**2907**	**0.54**	**19.6**	**14.3**
	9.5	1100	1489	2962	0.10	3.80	17.5
	10.2	1400	1271	3318	0.01	0.19	22.3
CO_2 flux over 100 or 50 µm distance (mmol $m^{-2}d^{-1}$)<br>Or µmol $cm^{-2}d^{-1}$<br>Or nmol $cm^{-2}h^{-1}$					51.5-103.0<br>5.15-10.3<br>217-433		

Note that data were based on all three species in Fig. 2 across their relative depth profiles plus the highest value from Ref. 1. Upper coelenteron is the first quarter of a profile where a larger pH and CO_3^{2-} increase were observed, usually at 300-500 µm. Bottom coelenteron is the last quarter of a profile, usually 0-500 µm above the calcifying fluid. CO_2 flux calculation into the calcification layer is calculated based on data in the two highlighted rows.

Supplementary Table 2. Measurement conditions for pH_{NBS} and CO_3^{2-} profiles for the coral *Orbicella faveolata*

	Date	Salinity	pH	T (°C)	TA (mmol kg^{-1})
pH1	04/22/2013	35.5	8.26	26.0	1.790
pH 2	05/07/2013	35.0	8.19	26.0	1.701
CO_3^{2-}	08/18/2013	35.0	8.17	26.0	1.700

Supplementary Table 3. Measurement conditions for pH_{NBS} and CO_3^{2-} profiles for the coral *Turbinaria reniformis*

	Date	Salinity	pH	T (°C)	TA (mmol kg^{-1})
pH1	01/09/2015	35.0	8.17	26.0	1.790
pH2	10/07/2012	35.0	8.06	26.0	2.040
CO_3^{2-} 1	07/11/2013	35.5	8.30	26.0	1.690
CO_3^{2-} 2	05/15/2013	35.0	8.24	26.0	2.087

Note that pH2 measurement was carried during nighttime (thus external tank seawater pH was low), but light was on during the microelectrode measurement. All others were collected during daytime.

Supplementary Table 4. Measurement conditions for pH_{NBS} and CO_3^{2-} profiles for the coral *Acropora millepora*

	Date	Salinity	pH	T (°C)	TA (mmol kg^{-1})
pH	09/20/2012	35.0	8.30	26.0	1.790
CO_3^{2-}	08/01/2013	35.0	8.10	26.0	1.611

Supplementary Table 5-part a. Carbonate system constraints and energetic requirements of the calcifying fluid models (low internal DIC)

external pCO_2 (ppm)		TA (umol/kg)	DIC (umol/kg)	pH(NBS)	[H]	HE/HI	ln(HE/HI)	Δ-Energy (%)	Δ-Energy (%)
400	External	2000	1756	8.14	7.213E-09			Ries 2011	Nernst eqn
600	External	2000	1824	7.99	1.014E-08				
900	External	2000	1884	7.84	1.449E-08				
2850	External	2000	2029	7.38	4.214E-08				
400	Internal	2600	1756	8.82	1.5E-09	4.8	1.57063	0.0	0.0
600	Internal	2600	1824	8.75	1.785E-09	5.7	1.73705	18.1	10.6
900	Internal	2600	1884	8.68	2.081E-09	7.0	1.94023	44.7	23.5
2850	Internal	2600	2029	8.52	3.047E-09	13.8	2.62706	187.6	67.3
400	Internal	4000	1756	9.93	1.167E-10	61.8	4.12384	0.0	0.0
600	Internal	4000	1824	9.83	1.465E-10	69.2	4.23711	12.0	2.7
900	Internal	4000	1884	9.75	1.794E-10	80.8	4.39156	30.7	6.5
2850	Internal	4000	2029	9.55	2.848E-10	148.0	4.99716	139.5	21.2
400	Internal	4900	1756	10.42	3.774E-11	191.1	5.25302	0.0	0.0
600	Internal	4900	1824	10.37	4.248E-11	238.7	5.47519	24.9	4.2
900	Internal	4900	1884	10.32	4.763E-11	304.1	5.71749	59.1	8.8
2850	Internal	4900	2029	10.18	6.57E-11	641.4	6.46371	235.6	23.0
400	External	2000	1756	8.14	7.213E-09				
600	External	2000	1824	7.99	1.014E-08				
900	External	2000	1884	7.84	1.449E-08				
2850	External	2000	2029	7.38	4.214E-08				
400	Internal	2804	1756	8.99	1.032E-09	7.0	1.94456	0.0	0.0
600	Internal	2709	1824	8.84	1.452E-09	7.0	1.9432	-0.1	-0.1
900	Internal	2602	1884	8.68	2.073E-09	7.0	1.94424	0.0	0.0
2850	Internal	2348	2029	8.22	6.043E-09	7.0	1.94226	-0.2	-0.1
400	Internal	3825	1756	9.79	1.607E-10	44.9	3.80379	0.0	0.0
600	Internal	3768	1824	9.65	2.259E-10	44.9	3.80405	0.0	0.0
900	Internal	3678	1884	9.49	3.227E-10	44.9	3.8042	0.0	0.0
2850	Internal	3256	2029	9.03	9.393E-10	44.9	3.80371	0.0	0.0
400	Internal	5545	1756	10.62	2.416E-11	298.5	5.69877	0.0	0.0
600	Internal	5163	1824	10.47	3.397E-11	298.5	5.69883	0.0	8.5
900	Internal	4883	1884	10.31	4.847E-11	298.9	5.69996	0.1	8.5
2850	Internal	4400	2029	9.85	1.411E-10	298.6	5.69922	0.0	8.5

Note that part a uses the exact same parameters as in Ries (2011)[1] under relatively low internal DIC conditions. Δ-Energy (%) (Ries 2011) follows the method as in Ries et al. (2011) but Δ-Energy (%) (Nerst eqn) follows the Nerst equation given in Ries et al (2011).

Supplementary Table 5-part b. Carbonate system constraints and energetic requirements of the calcifying fluid models (high internal DIC)

external pCO_2 (ppm)		TA	DIC	pH	[H]	HE/HI	ln(HE/HI)	Δ-Energy (%)	Δ-Energy (%)
400	External							Ries 2011	Nernst eqn
600	External								
900	External								
2850	External								
400	Internal	3900	3200	8.44	3.663E-09	2.0	0.67757	0.0	0.0
600	Internal	3900	3200	8.44	3.663E-09	2.8	1.01816	40.6	50.3
900	Internal	3900	3200	8.44	3.663E-09	4.0	1.3749	100.8	102.9
2850	Internal	3900	3200	8.44	3.663E-09	11.5	2.44277	484.3	260.5
0	Internal	6000	3200	9.53	2.977E-10	24.2	3.18761	0.0	0.0
600	Internal	6000	3200	9.53	2.977E-10	34.1	3.5282	40.6	10.7
900	Internal	6000	3200	9.53	2.977E-10	48.7	3.88493	100.8	21.9
2850	Internal	6000	3200	9.53	2.977E-10	141.6	4.9528	484.3	55.4
400	Internal	7350	3200	10.28	5.304E-11	136.0	4.91263	0.0	0.0
600	Internal	7350	3200	10.28	5.304E-11	191.2	5.25322	40.6	6.9
900	Internal	7350	3200	10.28	5.304E-11	273.1	5.60996	100.8	14.2
2850	Internal	7350	3200	10.28	5.304E-11	794.6	6.67783	484.3	35.9
400	External								
600	External								
900	External								
2850	External								
400	Internal	4206	3200	8.64	2.299E-09	3.1	1.14321	0.0	0.0
600	Internal	4063.5	3200	8.55	2.818E-09	3.6	1.28046	14.7	12.0
900	Internal	3903	3200	8.44	3.644E-09	4.0	1.38007	26.7	20.7
2850	Internal	3600	3200	8.17	6.825E-09	6.2	1.82049	96.9	59.2
400	Internal	5737.5	3200	9.39	4.06E-10	17.8	2.87722	0.0	0.0
600	Internal	5652	3200	9.35	4.474E-10	22.7	3.12085	27.6	8.5
900	Internal	5517	3200	9.28	5.197E-10	27.9	3.32769	56.9	15.7
2850	Internal	4884	3200	8.99	1.027E-09	41.0	3.71417	130.9	29.1
400	Internal	8317.5	3200	10.60	2.519E-11	286.4	5.65731	0.0	0.0
600	Internal	7744.5	3200	10.43	3.71E-11	273.3	5.61054	-4.6	-0.8
900	Internal	7324.5	3200	10.26	5.446E-11	266.0	5.58359	-7.1	-1.3
2850	Internal	6600	3200	9.87	1.349E-10	312.4	5.74417	9.1	1.5

Note that part b is under high internal DIC conditions with TA = 1.5X of the Ries and DIC = 3200 μmol kg^{-1}. Δ-Energy (%) (Ries 2011) follows the method as in Ries et al. (2011) but Δ-Energy (%) (Nerst eqn) follows the Nerst equation given in Ries et al (2011).

Supplementary Note 1

We calculated the relative energy changes of pH upregulation between different OA conditions as specified in Ries [1]. In the literature, Nernst potential across the membrane barrier is defined as (for example, equation 8 of Ries):

$E = (RT)/(nF) \ln(H_E/H_I)$,

where H_E and H_I are proton activity (or concentration) in the outside or internal solution respectively. Thus, energy change relative to 400 ppm external pCO_2 should be

$$\Delta E = \{E(pCO_2=x) - E(pCO_2=400)\}\ v/E(pCO_2=400) = \ln(H_E/H_I)_x/\ln(H_E/H_I)_{400} - 1 \qquad (1)$$

According to Table 1 in Reis (2011), he used the ratio of protons directly (i.e., H_E/H_I), not the natural logarithmic form.

In Supplementary Table 4-part a, we calculated both. In either way, energy cost is much smaller for scenario 2 (fixed H_E/H_I ratio) than for scenario 1 (fixed removal). In principle, energy change in scenario 2 should equal to 0 as the ratio H_E/H_I is fixed for all 4 external pCO_2 values. This is exactly reproduced in Supplementary Table 4-part a. Although there appears to be some numerical “noises or errors” in his calculation, the results in Ries Table 1 are generally consistent with the principle.

In Supplementary Table 4-part b, we multiplied the Ries (2011) TA values by 1.5 and set all DIC = 3200 μmol kg^{-1} (roughly a 2X seawater DIC scenario). While we did not try to generate exactly the same H_E/H_I ratios as in Reis, our ranges are quite close. The results show that energy cost of coral responding to ocean acidification due to atmospheric pCO_2 increase would be greater if the coral had a high internal DIC (except the very high pH or very high TA/DIC ratio case when the energy changes are similar for both low and high DIC cases).

Supplementary Note 2

To calculate the carbonate chemistry inside the coral polyp, the measured pH and CO_3^{2-} were used to calculate the other carbonate species using the following equations.

$$[CO_2] = [CO_{2aq}+H_2CO_3] = [H^+]^2*[CO_3^{2-}]/(K_1*K_2) \quad (2)$$

Note in the main text, $[CO_{2aq}+H_2CO_3]$ are simply given as $[CO_2]$. Here, $[CO_{2aq}]$ is the aqueous CO_2 which is >99.5% of the total molecular CO_2 (Ref. [2]). K_1 and K_2 are the first and second dissociation constants of carbonic acid[3].

$$pCO_2 = [CO_{2aq}+H_2CO_3] / K_H \quad (3)$$

$$[DIC] = [CO_2] + [HCO_3^-] + [CO_3^{2-}] = [CO_3^{2-}] ([H^+]^2/(K_1*K_2) + [H^+]/K_2 + 1) \quad (4)$$

$$C\text{-}Alk = [CO_3^{2-}] ([H^+]/K_2 + 2) \quad (5)$$

$$B\text{-}Alk = [B(OH)_4^-] = T_B*K_B/([H^+]+K_B), \quad (6)$$

$$TA = C\text{-}Alk + B\text{-}Alk \quad (7)$$

$$\Omega_{arag} = [Ca^{2+}][CO_3^{2-}]/K_{sp\text{-}arag}, \quad (8)$$

Here, C-Alk and B-Alk are carbon and boron alkalinity respectively. T_B is Total B concentration [4] and K_B is the dissociation constant of boric acid in seawater. $K_{sp\text{-}arag}$ is the solubility constant of aragonite.

Supplementary Note 3

Because salinity inside a coral polyp has not been measured, assuming seawater salinity is the general practice of the community[1,5,6]. Here S = 35 and T = 26°C were used for the calculation. We have also calculated DIC and TA by using a salinity range of ±5. At S=30, DIC is 0.8 to 11.8% higher and TA is 0.4 to 10.8% higher than those at S=35. At S=40, DIC is 0.6 to 8.8% lower and TA is 0.3 to 8.2% lower than those at S=35.

In addition, we performed the calculation using our typical pH(NBS) = 9.5 and $[CO_3^{2-}]$ = 1100 μmol kg^{-1} with several salinities and listed the results in the following table. At salinities 35, 17.5, and 9, we derived [DIC] = 1488.6, 1691.6, and 1951.3 μmol kg^{-1} respectively. Thus for the nature of the conclusions derived here (the range of CO_2 flux and whether internal DIC is 1X of seawater or 2X of seawater) and within a reasonable salinity range, such calculation differences are not important.

Supplementary References

1 Ries, J. B. A physicochemical framework for interpreting the biological calcification response to CO2-induced ocean acidification. *Geochimica et Cosmochimica Acta* **75**, 4053-4064, doi:http://dx.doi.org/10.1016/j.gca.2011.04.025 (2011).

2 Morel, F. M. M. & Hering, J. G. *Principles and Applications of Aquatic Chemistry*. 2nd edn, (John Wiley and Sons, 1992).

3 Millero, F. J., Graham, T. B., Huang, F., Bustos-Serrano, H. & Pierrot, D. Dissociation constants of carbonic acid in seawater as a function of salinity and temperature. *Marine Chemistry* **100**, 80-94 (2006).

4 Lee, K. *et al.* The universal ratio of boron to chlorinity for the North Pacific and North Atlantic oceans. *Geochimica et Cosmochimica Acta* **74**, 1801-1811, doi:http://dx.doi.org/10.1016/j.gca.2009.12.027 (2010).

5 Allison, N., Cohen, I., Finch, A. A., Erez, J. & Tudhope, A. W. Corals concentrate dissolved inorganic carbon to facilitate calcification. *Nat Commun* **5**, doi:10.1038/ncomms6741 (2014).

6 Hohn, S. & Merico, A. Modelling coral polyp calcification in relation to ocean acidification. *Biogeosciences* **9**, 4441-4454, doi:10.5194/bg-9-4441-2012 (2012).
